# Fast Determination of Auramine O in Food by Adsorptive Stripping Voltammetry

**DOI:** 10.1155/2019/8639528

**Published:** 2019-03-12

**Authors:** Thuong Nguyen Thi Kim, Thi Thu Bui, Anh Tuan Pham, Van Thang Duong, Thi Huong Giang Le

**Affiliations:** Department of Analytical Chemistry, Faculty of Chemistry, VNU University of Science, 19 Le Thanh Tong Street, Hanoi, Vietnam

## Abstract

The electrochemical behaviour of auramine O on the hanging mercury drop electrode has been investigated by cyclic and square wave voltammetry method. Reduction peak of auramine O was irreversible and adsorptive on the hanging mercury drop electrode. The optimal conditions were chosen to be Briton–Robinson buffer pH 9.0, accumulation potential −0.5 V vs. Ag/AgCl/KCl, accumulation time 60 s, pulse amplitude 250 mV·s^−1^, and frequency 50 Hz. At the optimum experimental conditions, the peak of the target analyte was sharp and asymmetric. The linearity of the peak current depending on the concentration ranged from 4.0 × 10^−8^ to 6.4 × 10^−7^ mol L^−1^. The limit of detection and limit of quantitation were 2.46 × 10^−8^ mol L^−1^ and 8.21 × 10^−8^ mol L^−1^, respectively. The recovery and relative standard deviation were 94.9% and 2.0% (*n* = 5). The developed method was successfully applied to determine auramine O in chicken samples with an appropriate sample preparation.

## 1. Introduction

**Figure 1:**
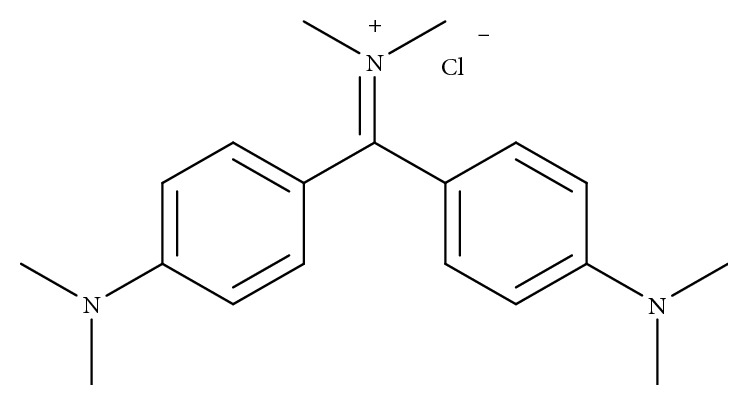
Chemical structure of AO.

Auramine O (AO), a diarylmethane dye, is an unauthorized food additive in Japan, the European Union, and United States due to its toxicity. AO is possibly causing cancer to humans and classified into Group 2B by the International Agency of Research on Cancer (Figure 1) [1, 2]. Parodi et al. [3] has reported that AO causes NDA damage in the liver, kidney, and bone marrow of rats and mice.

Toxicological data demonstrated in different animals show acute oral LD_50_ of 150–1500 mg/kg. Eye contact of AO may cause serious problems such as conjunctival edema, hyperemia, and purulent discharge, and more seriously total opacification. Patients who were long time exposure to AO may have the higher chance suffer from bladder cancer and lymphatic cancer [3, 4]. Many developing countries including the Philippines, India, Argentina, Malaysia, China, and Vietnam still use AO in food industry [5, 6]. Recently, despite the guide line on using AO, the use of AO in food industry has been rising in Vietnam. AO has been detected in various food and meat processes in Vietnam.

Several analytical methods based on many analytical platforms have been developed for the determination of AO in food, such as spectrophotometry [7, 8], liquid chromatography [6, 9], and fluorescence [10–12]. However, their drawbacks are cost, time-consuming (due to extra procedure e.g., preconcentration, multisolvent extraction, chromatographic separation). The voltammetry methods are highly sensitive, selective, fast, simple operation with low cost. On the other hand, adsorptive stripping voltammetry (AdSV) technique is suitable for analyzing electroactive organic compounds which can be adsorbed on the working electrode surface. Hanging mercury drop electrode (HMDE), which employing mercury as working electrode has a higher overvoltage, allows to extend the cathodic potential compared to solid electrodes e.g., paste carbon (PC), glassy carbon (GC), platinum (Pt), gold (Au). Moreover, the advantage of HMDE is having highly reproducible, smooth, and renewable surface.

In adsorptive stripping voltammetry method, accumulation potential (*E*_ad_), accumulation time (*t*_ad_), and pulse amplitude affect the sensitivity and selectivity of the method. The *E*_ad_ is applied to the working electrode and makes the electrode surface positively charged or negatively charged. For mercury electrodes, there is a point on the surface of the electrode that is not charged depending on the solution, which is called a “capillary electrical zero point.” Before that point is a positive charge and after that point is a negative charge, and the adsorption ability is affected by the applied electrode potential value. In addition, the adsorption process is influenced by other specific factors that have a chemical-specific affinity for Hg such as sulfur and nitrogen compounds. The AO is an organic compound containing nitrogen group, so it has the adsorptive ability on the surface of HMDE.

The concentration of the analyte substance on the surface of electrode is depended on accumulation time. Concentration of the target analyte is increased because of physical adsorption, electrostatic interaction, specific affinity of S-Hg and N-Hg, e.g., it will increase substance concentration on the surface electrode several times. Therefore, the amount of adsorption on the surface electrode will depend on the accumulation time. For too long, it will become multilayer, impeding the analysis process.

The challenge of developing an effective, low cost, and fast method for determination of AO in food is still essential. To our knowledge, it has not been found in the voltammetry methods published to determine AO in food to the moment yet. In this work, we present a cathodic adsorptive stripping square-wave voltammetry method combining with HMDE for highly selective and sensitive determination of AO in chicken meat.

## 2. Materials and Methods

### 2.1. Reagents and Instrumentation

All solutions were prepared with double distilled water (ddH_2_O). All reagents were analytical pure. Auramine O (85% HPLC grade) was obtained from the USA. A stock solution of 1.0 × 10^−3^ mol·L^−1^ AO was prepared in ddH_2_O. Standard solutions were prepared daily by dilution of the stock solution with ddH_2_O. Working solutions were measurement solutions containing AO and buffer solution. The stock solution was kept in the dark bottles immediately after preparation and stored at 4°C.

Britton–Robinson (BR) buffers of pH from 2 to 10.5, prepared from mixtures of 0.04 mol·L^−1^ acetic, orthophosphoric, and boric acids, were used as supporting electrolytes. The required pH of the buffer were adjusted with 0.2 mol·L^−1^ sodium hydroxide solution [13, 14].

All measurements were performed with a *μ*Autolab type III (Netherlands), interfaced to the electrode assembly 663 VA Metrohm (Switzerland), and controlled by software 757 VA. The three-electrode system consists of a HMDE (working electrode), an Ag/AgCl/KCl (reference electrode), and a glassy carbon rod (auxiliary electrode). A stirring rod provides the convective transport during the preconcentration step. Prior to measurement, a completely deuteration for solution in electrolysis cell with high-purity nitrogen for at least 5 min was required.

### 2.2. Analytical Stripping Procedure

Samples consist of 10 mL of pH 9.0 BR buffer, and several AO concentrations were introduced into the electrolysis cell. Prior to the recording the voltammogram, oxygen was removed by a pure nitrogen stream for 5 min. To adsorb AO on the surface electrode, an accumulation potential *E*_acc_ = −0.5 V (vs. Ag/AgCl/KCl_s_) was applied to the HMDE with accumulation time *t*_acc_ = 60 s while the solution was kept stirring. After that, the stirring rod was stopped for 5 s to allow the solution to become quiescent, and the analytes distributed evenly on working electrode surface. The voltammogram was recorded immediately by cathodic polarization using square-wave technique.

### 2.3. Preparation of Sample Solutions from Chicken Meat

All chicken samples were obtained from some markets in Hanoi. The chicken meat samples were finely ground; after that, we weighed exactly 1 g of sample and dissolved in 4 mL of a mixture of acetonitrile (ACN)-water (H_2_O) 50: 50 (v/v). The sample was then shaken for 10 min and centrifuged at 6000 rpm for 15 minutes. The supernatant was collected in a flask. The same procedure was applied with residual precipitates two times, and the supernatant liquid was also collected to flask. The flask contained supernatant which then was soaked in hot water (about 80 degrees C) and filtered using a 0.45 *μ*m filter. A volume of 3.0 mL of filtered solution was transferred to a 25.0 mL volumetric flask, added with 10 mL of pH 9.0 BR buffer, and made up to 25.0 mL with ddH_2_O.

### 2.4. Method Linearity and Recovery Assessment

With the optimized experimental conditions and instrumental parameters, we evaluated the developed method through linear range and recovery. We have constructed linearity calibration in standard solutions and spiked chicken meat samples from 4.10^−8^ to 64.10^−8^ mol·L^−1^ and from 5.0 to 30.4 *μ*g·g^−1^, respectively.

The accuracy of the method was tested by performing the recovery studies at different levels of standard solution added to the samples. We spiked the AO solution into chicken meat and treatment sample followed as described in Section 2.3:

(1) $$\begin{array}{c} \%\text{ recovery}=\;\;\frac{\left(C_{\text{AO}f}-C_{\text{AO}i}\right)\times 100}{C_{\text{AO}d}}, \end{array}$$

where *C*_AO*i*_ is the amount of AO found in the sample before addition of standard solution, *C*_AO*f*_ is the amount of AO found after addition of standard solution, and *C*_AO*d*_ is the amount of standard AO added.

### 2.5. Method Validation

To validate the results using adsorptive stripping voltammetry method, same spiked samples were sent the laboratory of Vincent certification and inspection joint stock Company. The Ultra Performance Liquid Chromatography-tandem Mass Spectrometry (UPLC-MS/MS) method was used to determine auramine O content in samples. UPLC conditions was performed on ACQUITY UPLC BEH C18 column (i.e., 2.1 mm × 50 mm; partical size 1.7 *μ*m, Water) using a multistep gradient elution with 5 mM ammonium acetate (pH 3.0 with formic acid) and methanol as the mobile phase [9]. The flow rate was 0.3 mL/min, and the column temperature was maintained at 35°C. The sample solution for UPLC was injected and analyzed in the ESI (+) mode with selected ion monitoring (SIM) using selected ion masses of *m*/*z* 268.24 for LC/MS detection.

## 3. Results and Discussion

### 3.1. Cyclic Voltammetry

**Figure 2:**
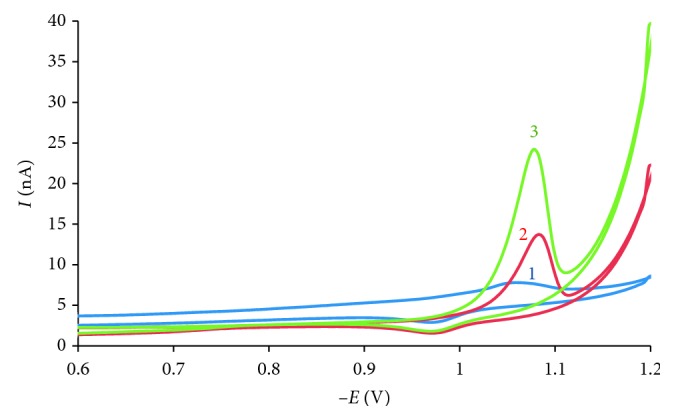
Cyclic voltammogram at scan rate 50 mV·s^−1^ and *E*_acc_ = 0 V of (1) blank sample (pH = 5.0, *t*_acc_ = 0 s); (2) 10^−6^ mol·L^−1^ AO solution (pH = 5.0, *t*_acc_ = 0 s); (3) 10^−6^ mol·L^−1^ AO solution (pH = 5.0, *t*_acc_ = 30 s).

Preconcentration time of cyclic voltammetry method was set for 0 and 30 seconds at the same accumulation potential of 0 V in pH 9 BR buffer to study the adsorptive character of AO on the surface of the working electrode. All setting conditions were the same in two cases. In terms of peak area, the peak of AO with preconcentration (curve 3) was higher than the one without preconcentration (curve 2), which indicated that AO was adsorptive on the electrode surface (Figure 2).

**Figure 3:**
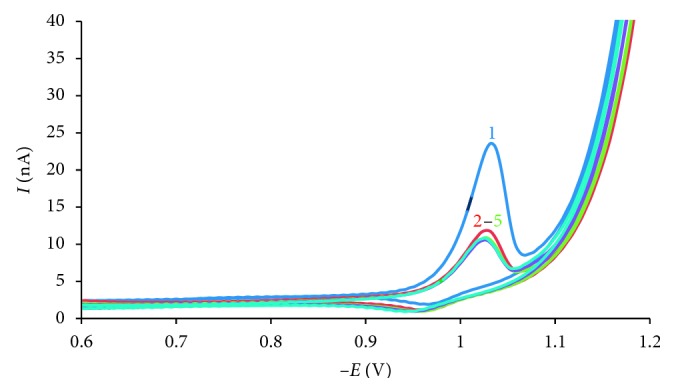
Cyclic voltammogram at scan rate 50 mV·s^−1^ and *E*_acc_ = 0 V for 10^−6^ mol·L^−1^ AO solution with *t*_acc_ = 30 s (1); (2–5) repetitive cyclic of (1) at the same mercury drop.

On the other side, the cyclic voltammogram was recorded 5 times with the same solution of 10^−6^ mol·L^−1^ AO, with the same mercury drop at scan rate 50 mV·s^−1^, *E*_acc_ = 0 V. The results (Figure 3) show that the repetitive cathodic peak current (curves 2, 3, 4, and 5) decreased rapidly compared to the first cathodic cycle, because of auramine O on the mercury electrode surface had been desorption when working electrode polarization.

The influence of pH on the AO cyclic voltammogram was also studied with various pH BR buffers in range of pH 5–9. The results show that a reduction peak was irreversible and when pH increased, the peak potential shifted to a more negative value, which indicated the involvement of protons in the electrode reaction [15].

The effect of scan rate on the reduction peak potential (*E*_pc_) and peak current (*I*_pc_) of AO on HMDE was examined by varying the scan rate from 10 to 700 mV·s^−1^.

**Figure 4:**
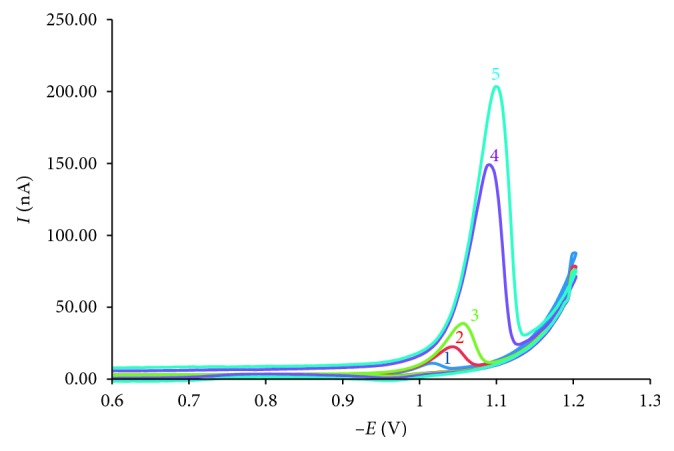
Cyclic voltammograms for 10^−6^ mol·L^−1^ AO in BR buffers of different scan rates (mV·s^−1^): (1) 10; (2) 50; (3) 100; (4) 500; (5) 700; *E*_acc_ = 0 V, *t*_acc_ = 30 s.

Figure 4 indicates the cyclic voltammograms of 10^−6^ mol·L^−1^ AO in BR buffer solution pH = 9.0 at a scan rate ranging between 10 and 700 mV·s^−1^. The electrode reaction was irreversible as shown by the lack of an oxidation peak in the cyclic voltammogram.

**Figure 5:**
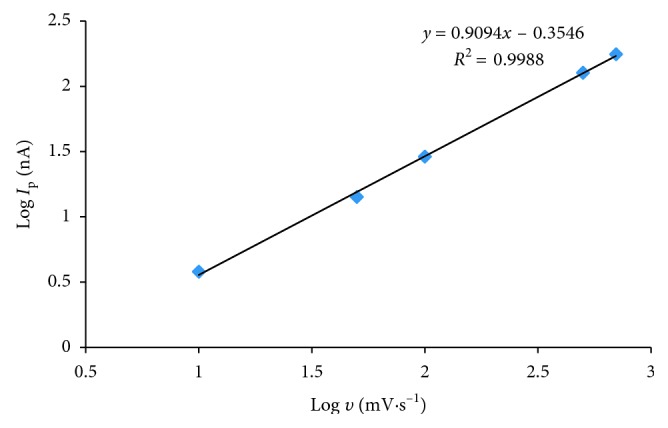
Cyclic voltammetry current (log *I*_p_) vs. log *ʋ* plot for 10^−6^ mol·L^−1^ AO : *E*_acc_ = 0 V, *t*_acc_ = 30 s scan rate from 10 mV·s^−1^ to 700 mV·s^−1^.

The peak current depended linearly on scan rates of 10–700 mV·s^−1^ according to the equation log *I*_p_ = 0.909 log *ʋ* − 0.35; *R*^2^ = 0.9988, *n*=5 (Figure 5). For such a relation, the slope value of 1.0 indicated an ideal surface reaction species, while the value of 0.5 was reaction kind in the solution [16]. Base on experimental results, the slope value of equation log *I*_p_ vs. log *ʋ* plot was 0.909 which showed that AO had strongly adsorbed on the surface of HMDE. Moreover, the irreversible reduction process of AO was confirmed by the shift of the peak potential (*E*_p_) to more negative values when the scan rate was increased [17].

### 3.2. Square Wave Voltammetry

**Figure 6:**
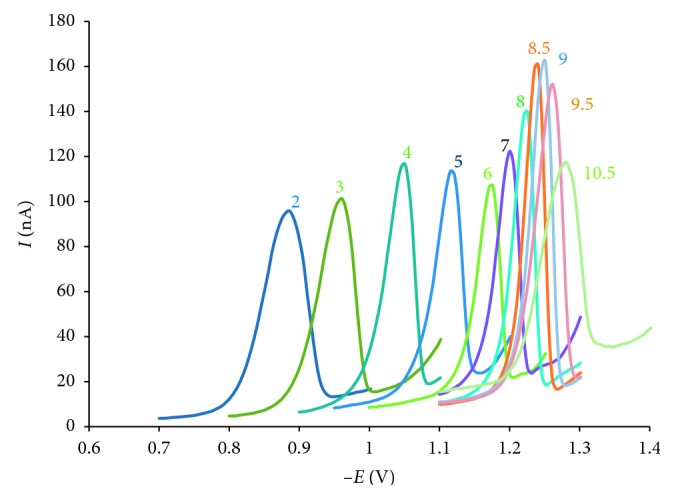
Adsorptive stripping voltammograms for 10^−7^ mol·L^−1^ AO in BR buffer of different pH 2.0–10.5; *E*_acc_ = −0.5 V, *t*_acc_ = 30 s, and scan rate 250 mV·s^−1^.

After accumulation of AO onto the surface of HMDE in different pH 2.0–10.5, the square wave voltammetry method was applied to scan tripping AO on the surface electrode. The results showed only one reduction peak at various pH. Clearly, reduction peak current and peak potential was affected by pH. The peak potential depended linearly on pH according to equation *E*_p_ = −0.06 pH − 0.72 (*R*^2^ = 0.97, *n*=11). It is expected due to the involvement of ion H^+^ in the electrode reaction process. When pH increased, the peak potential shifted to more negative values due to the decrease of H^+^ concentration which prevented the reduction process. However, peak current increased when pH values increased from 2.0 to 9.0 and reached a maximum at pH of 8.5–9.0. When pH was higher (9.5), then peak current decreased (Figure 6). Hence, a BR buffer of pH 9.0 was used as a supporting electrolyte for all further measurements.

**Figure 7:**
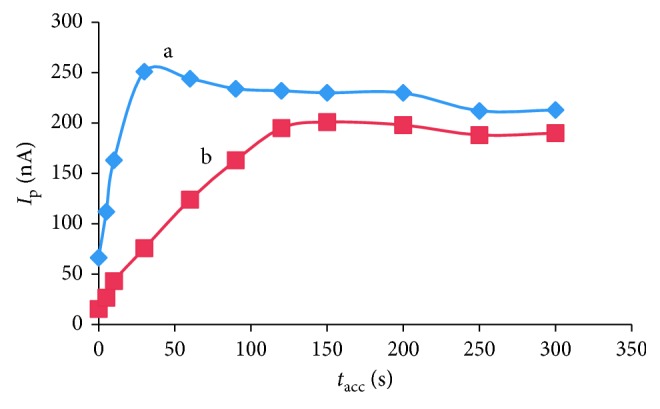
Effect of *t*_acc_ on the peak current (*I*_p_) of two different concentrations of AO: (a) 10^−6^ mol·L^−1^ and (b) 2.4 × 10^−7^ mol·L^−1^; *E*_acc_ = −0.5°V, scan rate 250 mV·s^−1^, *f* = 50 Hz.

**Figure 8:**
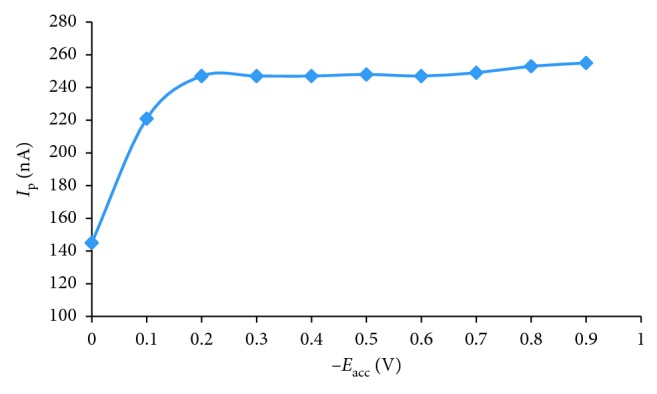
Effect of *E*_acc_ on the *I*_p_ for 10^−7^ mol·L^−1^ AO, *t*_ad_ = 60°s, *f* = 50 Hz.

Figure 7 shows the effect of *t*_acc_ and initial AO concentration on peak current. The peak current increased rapidly within a short *t*_acc_ period, and then decreased within long *t*_acc_ period, which indicated a fully coverage of the electrode surface. Especially, the peak current increased linearly in the range 0 s to 60 s and 0 s to 120 s accumulation time with the concentration 10^−6^ mol·L^−1^ and 2.4 × 10^−7^ mol·L^−1^ AO, respectively. So, with concentration of AO higher and lower than 10^−7^ mol·L^−1^, we chose 60 s and 120 s to accumulate AO on the surface of HMDE, respectively. We studied the dependence of the reduction peak current on the accumulation potential over the range −0.0 to −0.9 V for 10^−7^ AO after preconcentration for 60 s. The reduction peak current increased when accumulation potential changed from 0 V to −0.3 V; after that, the reduction peak current was not depended on the accumulation potential from −0.3 V to −0.9 V (Figure 8). Therefore, an accumulation potential of −0.5 V was chosen to determine AO in food samples.

**Figure 9:**
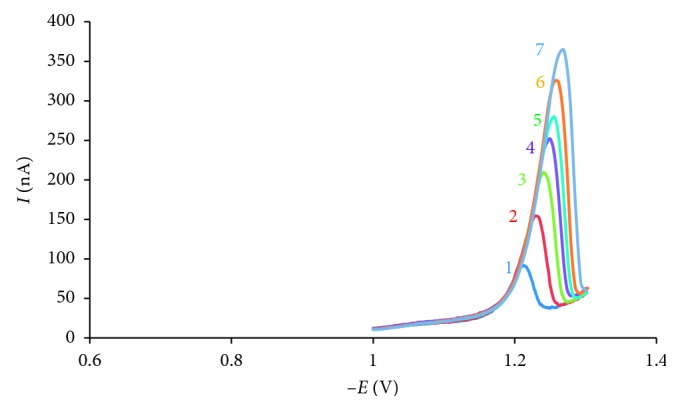
Voltammograms of 10^−7^ mol·L^−1^ AO in BR buffer depending on scan rate from 50 mV·s^−1^ to 400 mV·s^−1^ (1: 50 mV·s^−1^, 2: 100 mV·s^−1^, 3: 150 mV·s^−1^, 4: 200 mV·s^−1^, 5: 250 mV·s^−1^, 6: 300 mV·s^−1^, and 7: 400 mV·s^−1^), with conditions 10^−7^ mol·L^−1^ AO, pH = 9.0, *t*_acc_ = 60 s, and *f* = 50 Hz.

Reduction peak current depends on scan rate (*ʋ*); under the above optimized conditions, the reduction peaks current increased linearly with the scan rate in range of 50–400 mV·s^−1^ followed by the equation *I*_p_ = 0.67*ʋ* + 52.24 (*R*^2^ = 0.98, *n*=7) and peak potential shifted to more negative values when scan rate increased (Figure 9). For further measurements, a scan rate of 250 mV·s^−1^ was chosen.

**Figure 10:**
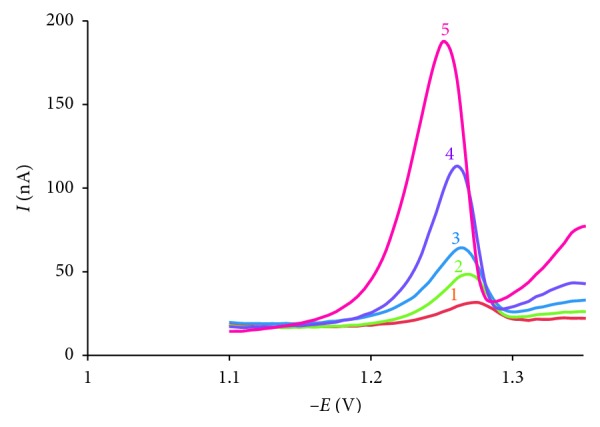
Voltammogram of AO in the range of 10^−8^ mol·L^−1^–6.4 × 10^−7^ mol·L^−1^ with conditions pH = 9.0, *E*_acc_ = −0.5 V, *t*_acc_ = 60 s, scan rate = 250 mV·s^−1^, *f* = 50 Hz. (1) 4.10^−8^ mol·L^−1^; (2) 12.10^−8^ mol·L^−1^; (3) 20.10^−8^ mol·L^−1^; (4) 32.10^−8^ mol·L^−1^; (5) 64.10^−8^ mol·L^−1^.

The optimal conditions for the analyse of AO were established as pH = 9.0, *t*_acc_ = 60 s, *E*_acc_ = −0.5 V, scan rate = 250 mV·s^−1^, and frequency = 50 Hz. Linear calibration for AO was obtained over the concentration range 4 × 10^−8^ mol·L^−1^ to 6.4 × 10^−7^ mol·L^−1^ (Figure 10).

The dependence of *I*_p_ on AO concentration (*C*_*x*_) was straight line according to equation *I*_p_ (nA) = 2.46 × *C*_*x*_ (×10^−8^ mol·L^−1^) + 2.89, *R*^2^ = 0.999, *n*=5.

The results (Figure 10) show that at the same *t*_acc_, when the concentration of the analyte in the solution increased, the concentration of the analyte on the surface of the electrode increased, and AO was reduced easier so that peak potential shifted to more positive values. This result was consistent with the results of investigating the effect of adsorption time; as the adsorption time increased, the concentration of the analyte on the surface of the electrode increased and peak potential moves toward the positive.

### 3.3. Validation of Method

To validate an analytical method for determinations AO before the extended analytical applications, we evaluated the limit of detection (LOD), limit of quantification (LOQ), and precision. From the calibration curves, we calculated LOD, LOQ with formula 3*S*_D_/*b* and 10*S*_D_/*b*, where “*S*_D_” is the standard deviation of the intercept and “*b*” is the slope of the calibration curve [18]. The LOD and LOQ of the method were 2.46 × 10^−8^ mol·L^−1^ and 8.21 × 10^−8^ mol·L^−1^, respectively.

Precision was determined by ten successive measurements of solution containing 3.2 × 10^−7^ mol·L^−1^ and 1.0 × 10^−7^ mol·L^−1^ of AO in pH 9 BR buffer using an accumulation time of 60 s at accumulation potential −0.5 V. The relative standard deviation was 1.24% and 1.67% with the concentration of 3.2 × 10^−7^ mol·L^−1^ and 1 × 10^−7^ mol·L^−1^ AO, respectively.

### 3.4. Applications

The proposed procedure was successfully applied to the determination of AO in chicken meat. The procedure for the AO analysis was followed as described in the sample preparation section. The quantitative of determination of the different concentration of AO in spiked chicken meat samples were carried out by using the AdSV. The linear calibration graphs for AO in chicken meat were obtained in concentration from 5.0 *μ*g/g to 30.4 *μ*g/g. The dependence of the peak current (*I*_p_) and the concentration of AO in spiked chicken meat was a straight line following the equation *I*_p_ = 3.42 × *C*_*x*_ (*μ*g·g^−1^) −9.31 (*R* = 0.991, *n*=6). The LOD and LOQ of AO in chicken meat was found at 1.0 *μ*g·g^−1^ and 3.2 *μ*g·g^−1^, respectively. The reliability of the method for the determination of AO in different spiked chicken meat samples was tested. The mean recovery of method for determination AO in spiked chicken meat was found to range from 84.8% ± 1.89 to 103.2% ± 1.28 (*n*=10 replicate measurements). From the obtained results, we applied this procedure to determine AO in chicken meat samples which were bought at Nguyen Cao, Khuong Dinh, Van Dien, Truong Dinh markets. The measured results showed that the method had not detected AO content in chicken meat samples bought at the market.

With spiked chicken meat samples, we determined AO content by adsorptive tripping voltammetry (AdSV) and Ultra Performance Liquid Chromatography-tandem Mass Spectrometry method (UPLC-MS/MS). The results obtained by both methods are summarized in Table 1.

As can be seen (Table 1), the results obtained with the AdSV are in good agreement with UPLC-MS/MS. However, the voltammetric method is simpler and faster and requires less expensive equipment than the chromatographic method.

## 4. Conclusion

We developed an adsorptive stripping voltammetry for the determination of auramine O in food. The electrochemical behaviour of AO was an irreversible process and adsorptive on the surface of HMDE. With the optimum conditions, the proposed method has high sensitivity, well repeatability, high precision and accuracy, and high recoveries from 84.8% to 103.2%. The method successfully applied to the fast determination of AO in chicken meat samples. The results are in good agreement with the Ultra Performance Liquid Chromatography-tandem Mass Spectrometry method.

## Figures and Tables

**Table 1:** Determination of AO in spiked chicken meat samples by AdSV and UPLC-MS/MS method.

Samples	Amount of spike AO (*μ*g/g)	AdSV assay^*∗*^, % found	UPLC-MS/MS, % found
1	6.08	94.6	80.1
2	9.12	83.4	84.8
3	24.3	102.7	98.3

^*∗*^Results are the means of three replicate determinations.

## Data Availability

The data used to support the findings of this study are included within the article.
